# Tick-Borne-Agents Detection in Patients with Acute Febrile Syndrome and Ticks from Magdalena Medio, Colombia

**DOI:** 10.3390/pathogens11101090

**Published:** 2022-09-24

**Authors:** Ruth Cabrera, Willington Mendoza, Loreth López-Mosquera, Miguel Angel Cano, Nicolas Ortiz, Valentina Campo, Yoav Keynan, Lucelly López, Zulma Vanessa Rueda, Lina Andrea Gutiérrez

**Affiliations:** 1 Grupo Biología de Sistemas, Escuela de Ciencias de la Salud, Facultad de Medicina, Universidad Pontificia Bolivariana, Medellín 050034, Colombia; 2 Grupo de Investigación en Salud Pública, Escuela de Ciencias de la Salud, Facultad de Medicina, Universidad Pontificia Bolivariana, Medellín 050034, Colombia; 3 Department of Internal Medicine, Medical Microbiology & Infectious Diseases and Community Health Sciences, University of Manitoba, Winnipeg, MB R3E 0J9, Canada

**Keywords:** bacterial zoonoses, molecular diagnostic techniques, serologic tests, Coxiella

## Abstract

Acute febrile illness (AFI) is a morbid condition with a sudden onset of fever with at least seven days of evolution, where no signs or symptoms related to an apparent infection have been identified. In Latin America, a high proportion of disease is typically due to malaria and arboviruses. However, among the infectious etiologies, tick-borne diseases (TBDs) should also be considered, especially in areas where people come into direct contact with these arthropods. This study aims to describe the etiology and epidemiology related to tick-borne agents in patients with AFI and the tick’s natural infection by agents of TBD in the rural tropical Magdalena Medio region in Colombia, and explore the factors associated with the presence of *Coxiella burnetii* infection. We conduct a cohort study enrolling 271 patients with AFI to detect the bacteria of the genera *Anaplasma, Ehrlichia, Coxiella, Rickettsia, Borrelia*, and *Francisella* through molecular techniques, and additionally evaluate the presence of IgG antibodies with commercially available kits. We also conduct tick collection in the patient’s households or workplaces for the molecular screening of the same bacterial genera. Seropositivity to IgG antibodies was obtained for all the bacteria analyzed, with *Francisella* being the most common at 39.5% (107/271), followed by *R. rickettsii* at 31.4% (85/271), *Ehrlichia* at 26.9% (73/271), *R. typhi* at 15.5% (42/271), *Anaplasma* at 14.4% (39/271), and *Borrelia* at 6.6% (18/271). However, these bacteria were not detected by the molecular techniques used. *Coxiella burnetii* infection was detected in 39.5% of the patients: 49.5% only by phase I and II IgG antibodies, 33.6% only by real-time PCR, and 16.8% had a concordant positive result for both techniques. A total of 191 adult ticks, 111 females and 80 males, were collected and identified as *Rhipicephalus sanguineus* s.l. and *Rhipicephalus microplus*. In the 169 adult ticks in which natural infection was evaluated, *Ehrlichia* spp. was detected in 21.3% (36/169), *Coxiella* spp. in 11.8% (20/169), and *Anaplasma* spp. in 4.7% (8/169). In conclusion, we identified the prior exposition to *Francisella, Anaplasma, Ehrlichia, Rickettsia, Borrelia*, and *Coxiella* in patients through serological tests. We also detected the infection of *C. burnetii* using molecular techniques. In the ticks, we identified bacteria of the genera *Coxiella, Anaplasma,* and *Ehrlichia*. These results suggest the importance of these zoonotic agents as possible causes of AFI in this region.

## 1. Introduction

Acute febrile illness (AFI) or acute undifferentiated fever is defined as a morbid state with the sudden onset of fever within less than seven days of evolution [1]. The differential etiological diagnosis for AFI is particularly challenging in tropical regions due to environmental conditions, socio-economic factors, and limited resources [2]. In addition, the great diversity of possible infectious etiologies may confound healthcare providers because most patients present non-specific symptoms, such as fever, fatigue, headache, and muscle ache [3]; hence, diagnosis relies on the index of suspicion and performing the appropriate diagnostic tests.

Zoonotic diseases are widespread among the infectious etiologies for AFI, including tick-borne diseases (TBDs). More than six out of every ten known infectious diseases can be spread from animals, and three out of every four new or emerging infectious diseases in people originate from animals [4]. The frequency of emerging vector-borne zoonoses has significantly increased during the last ten years [5]. Ticks, along with mosquitoes, are recognized as the main arthropod vectors of microorganisms transmitted to humans and domestic animals globally [6]. They are responsible for the transmission of many infectious agents: bacteria (*Borrelia, Anaplasma*), viruses (tick-borne encephalitis), and even parasites (*Babesia*, *Theileria*) [7].

Humans are accidental hosts in these diseases associated with ticks, and transmission usually occurs through hematophagous bites [8]. The pathogen may persist for a long time in ticks because it can be transmitted from stage to stage (trans-stadial transmission), from females to eggs (vertical transmission), and from tick to tick via the host (horizontal transmission), depending on the pathogen [9]. In a study published in 2022, 529 ticks of 7 different species (*Ixodes ricinus*, *Haemaphysalis concinna*, *Haemaphysalis punctata*, *Dermacentor marginatus*, *Haemaphysalis inermis*, *Dermacentor reticulatus*, and *Rhipicephalus bursa*) from North-Western Spain were found to be infected with *Rickettsia* spp., *Anaplasma phagocytophilum*, piroplasms, *Borrelia burgdorferi* s.l., and *C. burnetii* through specific PCR [10], proving the importance of the vectors in transmitting these microorganisms from animals to humans.

Additionally, a study in 2016 performed on 314 ticks and 626 blood samples from the Netherlands reported that half of the ticks removed from humans tested positive for *Borrelia burgdorferi* sensu lato, *Anaplasma phagocytophilum*, *Candidatus* Neoehrlichia mikurensis, *Rickettsia helvetica*, *Rickettsia monacensis, Borrelia miyamotoi,* and several *Babesia* species. In 16 blood samples, DNA was detected from *Candidatus* Neoehrlichia mikurensis, *Anaplasma phagocytophilum*, *Babesia divergens*, *Borrelia miyamotoi*, and *Borrelia burgdorferi* s. l. [11]. At present, the diversity of causes behind severe AFI is considered a leading cause of preventable deaths in low- and middle-income countries.

Additionally, it has been reported that most fatal cases of AFI with an infectious etiology could have been treated earlier and diagnosed adequately [12]. However, limited microbiological and epidemiological studies evaluate the transmission dynamics of these pathogens in Colombian agricultural tropical areas. This study aims to describe the etiology and epidemiology related to tick-borne agents in patients with AFI and the tick’s natural infection by agents of TBD in the rural, tropical Magdalena Medio region in Colombia. In addition, the study aims to explore the factors associated with *C. burnetii* infection.

## 2. Results

### 2.1. Demographic Characteristics of the Patients

A total of 271 patients with acute febrile syndrome were enrolled in the study. Most patients were male (64.9%), 81.9% residing in Puerto Berrio, and 54.6% in rural areas. In addition, 60.1% reported having pets, with dogs (59.8%) and cats (57.6%) being the most common companion animals in the participants’ residences. Additionally, 91.9% consumed products derived from raw milk, such as cheese or butter, and 87.8% reported being bitten by ticks.

### 2.2. Patients Follow-Ups

Of the 271 patients, 92 accepted the collection of a second sample 2 to 4 weeks following enrollment, and 121 received a telephone follow-up session at 6 months.

In the first follow-up session, 3.3% (3/92) reported being diagnosed with malaria and the same percentage with dengue. Among the other most reported conditions, we observed headache and general malaise in 18.5% (17/92) of the patients. For *C. burnetii*, 22.8% (21/92) tested positive by qPCR, and only 9.5% (2/21) remained positive. In serology, 31.5% (29/92) presented phase I and/or II IgG antibodies, with 65.5% (19/29) presenting as positive in the second sample.

At the telephone call at 6 months, 39.5% (47/121) reported new consultations to the hospital for similar symptoms, 60.5% (72/121) reported headaches, 51.3% (61/121) fatigue, and 42.9% (51/121) fever. Furthermore, 2.5% (3/121) died.

### 2.3. Serological Evidence of Previous Exposure and Molecular Detection

Seropositivity to IgG antibodies was obtained for all the bacteria analyzed, with the *Francisella* genera being the most common at 39.5% (107/271), followed by *R. rickettsii* at 31.4% (85/271), *Ehrlichia* genera at 26.9% (73/271), *R. typhi* at 15.5% (42/271), *Anaplasma* at 14.4% (39/271), and *Borrelia* genera at 6.6% (18/271). The municipality of residence; the presence of cats, hens, sheep, and horses in the residence; and direct contact with animals in the workplace, among others, were identified as possible associated factors to previous exposure to these bacteria (Table S1). None of these bacteria were detected by the molecular techniques used in the current study.

In the *Coxiella burnetii* test, this microorganism was detected in 39.5% of the patients: 49.5% only by phase I and II IgG antibodies, 33.6% only by real-time PCR, and 16.8% had a concordant positive result using both techniques. This microorganism was detected in 40.3% of males vs. 37.9% of females, in addition to 50% of the people who reported being veterinarians or farmers and 61.9% of the patients who reported abortions of animals at work (Table 1).

Among the most reported clinical signs and symptoms in these patients were fatigue in 86% (92/107), chills in 89.7% (96/107), and headache in 90.7% (97/107) (Table 2).

### 2.4. Tick Data

Fifteen homes and one workplace of the patients who agreed to participate in the entomological screening were visited. A total of 61 people and 138 domestic animals were examined by the visual inspection technique. A total of 191 adult ticks, 111 females and 80 males, were collected. Thirteen canines (*Canis lupus familiaris*) and two bovines (*Holstein*) were infested with ticks. Then, the collected ticks were identified as *Rhipicephalus sanguineus* s.l. (93 females and 80 males), all collected from domestic dogs in seven AFI patient residences and one from a workplace. *Rhipicephalus microplus* (18 females) were collected from two bovines sampled in a patient’s house (Table 3).

A total 3 of out of the 16 sites included in the entomological screening had green areas, gardens, or paddocks to perform the dragging technique; those 6 areas were examined, and we obtained a positive result in 1. Ninety-five larvae of ticks were obtained, while nymphs were not found using the dragging method.

Genomic DNA was extracted from the complete body of 169 adult ticks; 16 adult ticks were stored in ethanol to support species identification, and 6 voucher specimens were deposited in the entomological collection of Tecnológico de Antioquia-CETdeA Colombia. The molecular confirmation of tick species based on the COI gene analysis correlated with the previous identification by morphological keys identifying the ticks *as Rhipicephalus sanguineus* sensu lato ‘tropical lineage’ and *Rhipicephalus microplus*. In the 169 adult ticks in which natural infection was evaluated, the bacteria of the genus *Ehrlichia* were detected in 21.3% (36/169), *Coxiella* in 11.8% (20/169), and *Anaplasma* in 4.7% (8/169). In the ticks identified as *R. sanguineus* s.l., these four genera were observed, while in those identified as *R. microplus*, only *Ehrlichia* and *Anaplasma* spp. were identified (Table 3). By the BLASTn analysis of the sequences obtained from these bacteria, one had an identity percentage range of 99–100% with uncultured *Ehrlichia* sp. (GenBank^®^ code: OM47536), one with *Anaplasma marginale* (GenBank^®^ code: OM475362), and two with *Anaplasma platys* (GenBank^®^ codes: OM475363 and OM475364).

### 2.5. Molecular Phylogenetic Analysis of 16s rRNA Sequences of the Coxiella Genus

**Figure 1:**
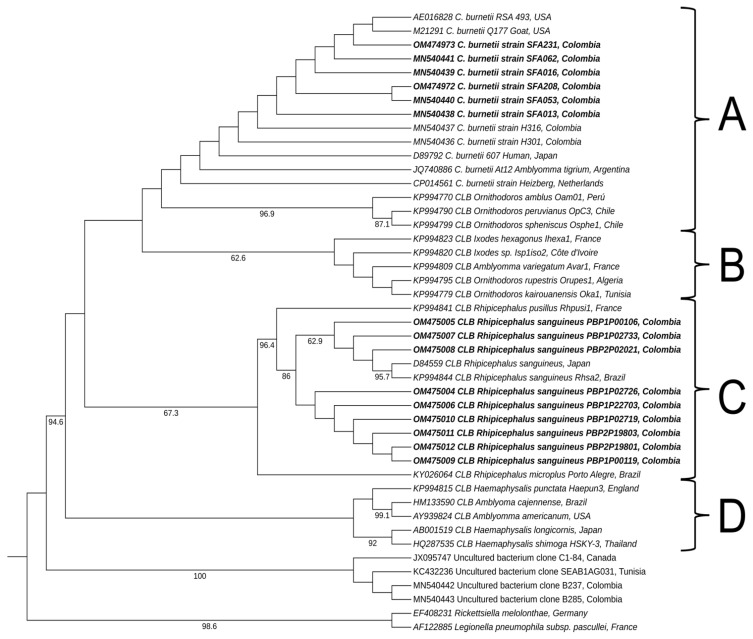
Phylogenetic tree constructed using maximum-likelihood based on partial sequences of *16S rRNA* for the *Coxiella* genus. All four clades previously reported for the *Coxiella* genus [13] are labeled with letters A to D. The *C. burnetii* group is included within clade A. The labels for each reference sequence include GenBank accession numbers and specify the country of origin. Its branches have numbers indicating bootstrap support (out of 1000 repetitions), ranging from 60% to 100%. The sequences obtained for the present study are displayed in a bold font. CLB: *Coxiella*-like bacteria.

BLASTn revealed that the sequences belonging to samples collected from six patients had high identity percentages (99–100%) of *C. burnetii* (GenBank^®^ codes: MN540438, MN540439, MN540440, MN540441, OM474972, and OM474973) and were grouped within A clade in the phylogeny analysis. In this group, this species had been previously described within the *Coxiella* genus [13]. The sequences collected from nine positive ticks showed the highest identity percentages, ranging from 98% to 100%, with *Coxiella*-like endosymbiont from *Rhipicephalus sanguineus* (GenBank^®^ codes: OM475004, OM475005OM475006, OM475007, OM475008, OM475009, OM475010, OM475011, and OM475012). Consistent with the molecular phylogeny analysis results, the sequences obtained from ticks were grouped in clade C (Figure 1).

### 2.6. Factors Associated with C. burnetii Detected in Patients with AFI

In the molecular screening of patients with AFI, *C. burnetii* was the only bacterium detected, so this study explored the possible factors associated with *C. burnetii* infection. The factors associated with an increase in *C. burnetii* detection frequency were type of health insurance, any animal that had abortions in the workplace, and pets allowed to sleep inside the residence. Those with lower frequencies identified similar symptoms in family members and the presence of chills (Table 4).

## 3. Discussion

In the current study, the IgG serology results identify prior exposure to all tick-borne agents studied among patients presenting with AFI in the Magdalena Medio region. However, molecular detection was observed only for the bacteria *C. burnetii*, suggesting it plays a role as a potential etiological agent of AFI in the Colombian Magdalena Medio region. We previously determined that *C. burnetii* infection was confirmed in livestock farmers from this region [14].

Regarding the other bacteria evaluated, 39.5% were positive for IgG antibodies against *F. tularensis*, which is high compared to other countries. A cross-sectional study conducted in Jordan identified, through ELISA, this microorganism in 7.7% of the 828 people evaluated. However, no convalescent-phase serum was analyzed, and the people did not present clinical symptoms of the disease [15]. Furthermore, in this same study, it was reported that those owning a small ruminant had 1.86 (95% CI: 1.02–3.40) greater odds for seropositivity than individuals who did not own a small ruminant, and those practicing horticulture had 2.10 (95% CI: 1.20–3.66) greater odds for seropositivity than individuals who did not practice horticulture [15]. Although we did not perform multivariate analyses as we could not confirm or exclude acute infection by these bacteria, the municipality of residence of the patients and animal abortions might be associated with the presence of this microorganism (Table S1).

Q fever is among the global priority bacterial zoonoses, including anthrax, brucellosis, leptospirosis, plague, salmonellosis, and zoonotic tuberculosis. However, it is still ignored and under-reported in most of the world [16]. *C. burnetii* causes Q fever, a zoonosis prevalent in all countries except New Zealand [17]. The bacterium’s hosts are broad, mainly including domestic and wild mammals, birds, and arthropods [18,19]. However, domestic ruminants, primarily cattle, sheep, and goats, are considered as essential reservoirs and transmission sources for humans [20].

One of the associated factors for detecting *C. burnetii* is that any animal that has had an abortion in the patient’s workplace (aPR: 1.81, 95% CI: 1.44–2.28). This bacterium can be recovered and identified in the placentas of infected animals, both from abortions and natural births [21]. According to the previous studies, the estimated bacterial load in an infected animal’s placenta can be as high as 10^9^ bacterial cells per gram of tissue [22]. As the estimated infectious dose through the inhalation of aerosols is slightly higher than one bacterial cell [23], people’s exposure to these highly infectious animal products represents an increased risk of exposure and transmission of this bacterium. Furthermore, suppose several herds are simultaneously affected in a wide geographic area; in that case, it can increase abortion rates in animals, increasing the probability of infection and the occurrence of human outbreaks [24].

Detecting *C. burnetii* in symptomatic and asymptomatic people is vital because there is no single management strategy. In countries where reporting this microorganism is mandatory, acute Q fever is defined by clinical symptoms and laboratory criteria [25]. This situation leads to underestimating primary infection cases by *C. burnetii*, without monitoring asymptomatic people, possibly increasing complications that are not related to the severity of the primary infection, but mainly to immunology factors [25]. Additionally, although this infection during pregnancy is frequently asymptomatic and underreported as it is not part of the screening tests of prenatal care, it can lead to severe obstetric complications, such as fetal death and malformations [26].

Regarding the clinical signs and symptoms identified in the present study, it is known that acute Q fever usually presents as a flu-like illness, with a sudden onset of high fever, which can last for more than 15 days and is frequently associated with myalgia and headaches [17,27]. In a study that followed-up 85 patients with the acute form of this disease, the main symptoms were fever, fatigue, headache, cough, myalgias, and arthralgias [28]. As it can be observed, this clinical presentation is non-specific and misleading, so physicians should request the specific detection of this microorganism in patients with acute febrile syndrome who present risk factors, such as direct contact with animals and a history of tick bites either in their residence or at work.

Goats and sheep are frequently identified as a source of Q-fever outbreaks in humans [29,30], as was the case in the Q-fever outbreak in the Netherlands in 2007–2010, where more than 4500 people were infected [31]. These infected animals can release high levels of *C. burnetii* into the environment during parturition. Other documented routes of elimination include raw milk, feces, urine, and saliva [32,33]. Previous studies have shown that cattle, goats, and horses can be potential reservoirs for *C. burnetii* and play an essential role in transmitting the infection. Specifically, a study conducted in Korea, where 592 blood samples were collected from animals, identified this microorganism in 22.7% of goats, 16.4% of dairy cattle, 15.2% of beef cattle, 6.0% of Boer goats, and 5.2% of horses [34], suggesting that these mammals may play an important role as reservoirs for this microorganism. Moreover, other studies have described *C. burnetii* seropositive individuals associated with rural residences, with an alleged relationship with the livestock number, finding the bacteria in 4.8% of the samples evaluated [35].

The clinical manifestations of Q fever are non-specific so the disease can be confused with other conditions, such as those caused by the influenza virus, dengue fever, malaria, leptospirosis, and hantavirus, among others. In 2016, Cortés et al. reviewed the most frequent diseases associated with the acute febrile syndrome to guide the general practitioner and specialist in providing a reasonable and rational approach to this syndrome [1]. Based on our results, we considered it essential to include *C. burnetii* in the list of differential diagnoses for people with acute febrile diseases, mainly in patients with risk factors for acquiring the infection.

We confirmed the usefulness of the COI gene sequence for the molecular identification of ticks by demonstrating that the morphological and molecular identifications of adult ticks of the species *Rhipicephalus sanguineus* s.l. and *Rhipicephalus microplus* were 100% congruent, confirming that the COI gene is one of the most valuable and informative markers for the identification of ticks [36,37,38]. The DNA of *Ehrlichia* spp. (16.7%), *Anaplasma* spp. (1.2%), and *Coxiella* spp. (11.6%) were detected in ticks of the species *R. sanguineus* s.l. from domestic dogs; *Ehrlichia* (38.8%) and *Anaplasma* (33.3%) were detected in ticks of the genus *R. microplus* recovered from the cattle of one participant. These results are congruent with the work of other authors on these tick species around the world [39,40] and in South America [41,42,43]. Additionally, these species are anthropophilic. In a study performed in Greece, 537 ticks were removed from humans by medical staff; all ticks belonged to three genera in the Ixodidae family: *Rhipicephalus* (469/519, 90.4%) with four species (*R. sanguineus, R. turanicus, R. bursa*, and R. [*Boophilus*] *annulatus*), *Hyalomma* (39/519, 7.51%) with three species (*H. marginatum, H. rufipes*, and *H. anatolicum*), and *Ixodes* (11/519, 2.12%) with two species (*I. rinicus* and *I. gibbosus*) [44]. This study suggested that humans that were in constantly around animals were at risk of becoming infected with TBD.

As a limitation of the present study, a second sample was only obtained from 33.95% of the patients (92/271), so they could not be analyzed as diagnostic tests. However, a serological fingerprint was detected in the patients from this area, which makes it essential to conduct studies that search for these microorganisms as causes of disease. This study presented an exploratory approach to the potential risk factors associated with *C. burnetii* infection; therefore, further studies must evaluate and confirm these findings.

Finally, conducting epidemiological studies that help to detect, through diagnostic techniques, the presence of zoonotic bacteria and specifically *C. burnetii* in biological samples, such as milk, vaginal swabs, and meat, among others, from animal hosts, such as goats, sheep, horses, and chickens, in addition to evaluating natural infections caused by these microorganisms in the adult and immature forms of ticks found in these animals, will help us to encourage health promotion and prevention strategies, including food safety in populations that depend on these types of production systems, and thus mitigate the impact of both on health and the economy that these microorganisms can affect in Colombia.

## 4. Materials and Methods

### 4.1. Area of Study, Patients, and Data Collection

**Figure 2:**
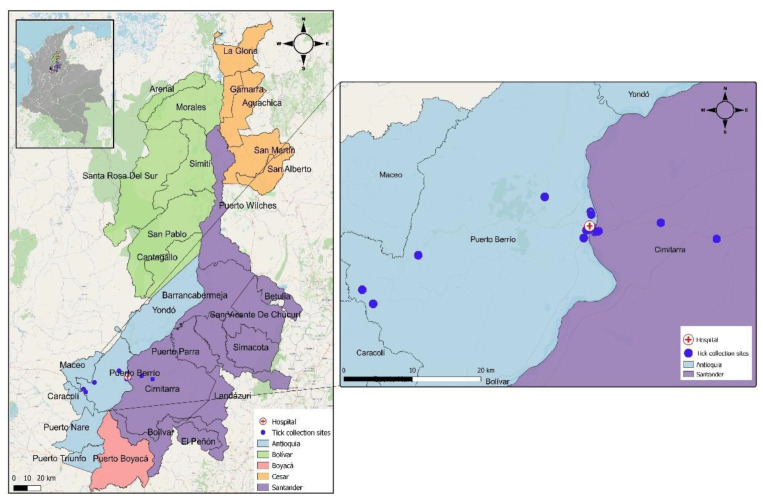
The geographic location of the 16 sites where tick collection was performed and the hospital of the second level of complexity from the Magdalena Medio region.

This cohort study enrolled patients who presented themselves for outpatient consultations, emergency visits, or hospitalizations in a hospital of the second level of complexity in the Magdalena Medio region between November 2018 and January 2020. This healthcare institution receives patients from various regional municipalities, such as Puerto Triunfo, Puerto Nare, Caracolí, San Roque, Maceo, and San José del Nus. It has an average monthly care rate of 2500 to 3000 patients in outpatient, 2700 in emergency, and 450 in hospitalization services [45]. It is located in the municipality of Puerto Berrio in Magdalena Medio (Antioquia, Colombia), where the temperature ranges from 32 to 43 °C, relative humidity is 53%, and the altitude ranges from 125 to 150 m.a.s.l. (Figure 2) [46].

A Total of 271 patients over 18 years of age were recruited who agreed to participate in the study and signed the informed consent and presented fever (oral temperature ≥ 38) lasting less than 2 weeks and 1 or more of the following symptoms: rash or hemorrhage, or lower respiratory tract infection, or jaundice or lymphadenopathy.

Patients were excluded if they had HIV category C (any opportunistic infection) or 3 (≤200 CD4 cells), consumed antibiotics for more than 72 h in the last 8 days, received treatment with steroids (prednisone ≥ 0.3 mg/kg/day for 3 weeks or more, or ≥1 mg/kg/day for ≥7 days) or cytostatics (except methotrexate at low doses: ≤15 mg/week), known neoplasms (except basal cell and thyroid carcinomas), granulocytopenia < 500 cells/mm^3^, a cough or expectoration for more than 15 days, kidney or liver failure, a history of trauma or previous surgery in the last 6 months, fever attributed to antibiotics, diarrhea as an initial and primary symptom, snakebites or acute poisoning, rhinitis, sinusitis, otitis, tonsillitis, or exclusive symptomatology of the upper respiratory tract.

At baseline, a structured questionnaire administered by the field team collected the sociodemographic characteristics, and the epidemiological and clinical aspects of each participant, including tick, animal, and food exposures in the workplace or at home, and other personal and environmental risk factors that were reported to be associated with TBD. In addition, a blood sample was obtained for acute serology and molecular diagnosis.

Each participant underwent a first follow-up visit between 2 to 4 weeks following enrollment for information about the evolution of the symptoms, and a second blood sample for molecular detection was collected. Additionally, each patient received a follow-up call at six months and was asked about recurrent febrile episodes, symptoms, and clinical diagnoses since the enrollment.

#### 4.1.1. Blood Sample Collection

Venous blood was obtained from each participant using a vacuum system. The samples were preserved and transported to the laboratory using sterile vacuum tubes, anticoagulant (for DNA extraction), and without anticoagulant (for serum separation).

#### 4.1.2. Molecular Detection and Serological Antibody IgG Analysis

Genomic DNA was extracted from each participant’s blood sample using QIAmp DNeasy Blood & Tissue (Qiagen, Valencia, CA, USA). The concentration obtained was evaluated in a Nanodrop 2000 (Thermo Scientific, Waltham, MA USA), and the DNA was stored at −20 °C until processing. All genomic DNA samples obtained from patients underwent a quality check (absence of amplification inhibitors) with real-time PCR amplification of the GAPDH enzyme gene using the primers GAPDH_F: 5′- TGG GTG TGA ACC ATG AGA AG -3′ and GAPDH_R: 5′- GCT AAG CAG TTG GTG GTG C -3′ [47]. The primers and probes for the molecular identification of the bacterial genera under study were used according to the conditions previously described (Table S2) [48,49,50,51,52,53]. Commercially available DNA controls Amplirun^®^ *Rickettsia conorii*, Amplirun^®^ *Borrelia burgdorferi*, Amplirun^®^ *Francisella tularensis,* and Amplirun^®^ *Coxiella burnetii* were used as positive controls; additionally, genomic DNA extracted from the Am13Vi-la strain of *Anaplasma marginale* provided by the Germplasm Bank of the Colombian Agricultural Research Corporation (AGROSAVIA), and genomic DNA from *Coxiella burnetii*, *Rickettsia rickettsii*, and *Ehrlichia canis* strains supplied by the Instituto de Investigaciones Biológicas del Trópico de la Universidad de Córdoba were used as positive controls according to each protocol. Milli-Q^®^ Type I (Merck, Darmstadt, Germany) water was used as a negative control.

The presence of IgG antibodies was determined through commercially available kits to test previous exposure (Table S2). The selected serological kits were implemented following the recommendations of the Centers for Disease Control and Prevention (CDC) for the detection of rickettsiosis (*Rickettsia* spp.), anaplasmosis (*Anaplasma* spp.), ehrlichiosis (*Ehrlichia* spp.) [54], Q fever (*Coxiella burnetii*) [25], borreliosis (*Borrelia burgdorferi*) [55], and tularemia (*Francisella tularensis*) [56].

### 4.2. Field Collection of Ticks and Species Identification

Tick collection was conducted in the patients’ homes or work environments, with a history of tick bites and positive results from molecular screening for *C. burnetii* to identify tick species distributed in the zone. We evaluated the natural infection of ticks by the microorganisms under study.

The specimens collected in this study were approved by the National Environmental Licensing Authority (ANLA, Spanish acronym): Collection Framework Agreement granted to Universidad Pontificia Bolivariana through resolution 744 - 26 July 2016. Preserved voucher specimens were deposited in the entomological collection Tecnologico de Antioquia-CETdeA (Medellín, Colombia). The dataset was uploaded to SiB Colombia’s data portal (https://sibcolombia.net/, accessed on 23 June 2021). Tick tracking was performed by physically inspecting the patient’s house (or the workplace of a patient dedicated to livestock), environment, and domestic animals present. The fieldwork was performed through intra-house collection.

Additionally, a peri-domiciliary collection was performed on a perimeter of 10 m around the house [57]. The collection of adult ticks was conducted by the visual inspection of an animal’s tail, loin, neck, and chest, and the extraction by hand of the adult ticks with a size larger than or equal to 3 mm [58,59,60]. Adult ticks were collected from domestic animals from December 2018 to February 2019, and individually stored in bottles with 95% ethanol [61]. The taxonomic identification of adult ticks was performed by evaluating each specimen using taxonomic keys based on the morphology described in the literature for genus and species [62,63,64]. Genomic DNA was extracted from each adult tick using the previously described protocol [65]. The molecular typing of a subset of the collected adult specimens was performed to confirm the morphological identification. Each adult-tick DNA sample was amplified by PCR using specific primers for a fragment of the cytochrome oxidase I (COI) gene (655–680 bp) with the primers LCO1490: 5′- GGT CAA CAA ATC ATA AAG ATA TTG G -3′ and HCO2198: 5′- TAA ACT TCA GGG TGA CCA AAA AAT CA -3′ [66], and COI sequences were bidirectionally obtained.

A white flannel (1 m^2^) was horizontally dragged through patios, gardens, and paddocks to gather non-parasitic tick forms. The attached ticks were removed from the flannel with a lint brush and placed in resealable bags with silica gel [67] until they were counted under a stereomicroscope (Nikon SMZ1270, Tokyo, Japan).

#### Molecular Detection of Natural TBD Infection in Ticks

For the molecular detection of natural infection by the bacteria of the *Anaplasma, Ehrlichia, Coxiella, Rickettsia, Borrelia*, and *Francisella* genera, the same protocols and primers used for molecular detection in patients with acute febrile syndrome were used to test all ticks’ DNA genomic samples (Table S2).

### 4.3. Data Analysis

#### 4.3.1. Molecular Phylogenetic Analysis for Coxiella Genus

The DNA sequences we obtained were edited, assembled, and aligned using the Geneious^®^ 9.1.2 program (Wellesley St, Auckland, New Zealand). For the molecular phylogenetic analysis, samples that were positive for the bacterium of the *Coxiella* genus, obtained through nested PCR (Table S2) [68], were used. This analysis was conducted based on the maximum-likelihood method and the PhyML plugin, available via the Geneious^®^ software [69]. During these analyses, the general time-reversible model was used, with gamma distribution (G) and the proportion of invariable sites (I) selected by the jModelTest 2.1 tool [70]. The robustness of the phylogenetic tree was estimated using bootstrap analysis with 1000 copies. *Legionella pneumophila* subsp. *pascullei* and *Rickettsiella melolonthae* were included as external groups. Tree appearance was edited using the Interactive Tree Of Life (iTOL) version 4.4.2 [71].

#### 4.3.2. Tick Data

The morphological tick-species identification was complemented with molecular analyses performed in the COI species database in the Barcode of Life Data System (BOLD)v4 using the following options: animal identification (COI) and species level barcode records.

The absolute and relative frequencies of tick adult species observed in each host were calculated (Microsoft Excel, Microsoft Corporation, Redmond, USA). Furthermore, each sample collection site counted the number of non-parasitic tick forms (larvae and nymphs) obtained in the gardens and paddocks.

#### 4.3.3. Descriptive and Inferential Statistical Analysis of Associated Factors

The real-time PCR performed to detect *C. burnetii* plus the serologic detection of phase I and II IgG antibodies were defined as the outcome variables. When analyzing the factors associated with *C. burnetii* infection, the crude prevalence ratio (cPR) and adjusted prevalence ratio (aPR) and their corresponding confidence intervals (CIs) at 95% were estimated with a generalized linear model with a Poisson distribution and logarithmic link, with adjustment by cluster with the variable municipality of residence in Stata^®^ software (Lakeway Drive, TX, USA). The variables were included in the multivariate model because they met the statistical criterion of a *p*-value of < 0.25 after performing bivariate analysis or because they played an essential role in disease transmission or were risk factors.

Relative and absolute frequencies were calculated for qualitative variables using SPSS^®^ statistical software (SPSS^®^ Inc., Armonk, NY, USA).

## 5. Conclusions

In this study, we identified through serological tests the previous exposition to *Francisella, Rickettsia* spp., *Ehrlichia* spp., *Anaplasma* spp., and *Borrelia* spp. Additionally, *C. burnetii* was identified in 39.5% of the patients either by serology or molecular techniques, considering the type of health insurance, any animal that had an abortion in the workplace, and pets allowed to sleep inside the residence as the factors associated with the infection. The ticks collected were identified as *Rhipicephalus sanguineus* s.l. and *Rhipicephalus microplus* and the bacteria of the genus *Ehrlichia* spp., *Coxiella* spp., and *Anaplasma* spp. by PCR. The phylogenetic tree constructed with sequences of 16S rRNA for the *Coxiella* genus showed that the sequences obtained from the patients were *C. burnetii* and from ticks were *Coxiella*-like endosymbionts from *Rhipicephalus sanguineus.* This project integrated human- and animal-health elements, showing the importance of these microorganisms and the challenges of confirming the etiology of AFIs, and suggested that *C. burnetii* should be considered as a potential etiology of AFIs in this region.

## Figures and Tables

**Table 1:** Demographic and epidemiological characteristics describing patients with acute febrile syndrome according to a positive result in any test used to detect *Coxiella burnetii*.

Demographic/Epidemiological Characteristics	n/N (%) ^1^	*p*-Value
Sex		
Male	71/176 (40.3)	0.694
Female	36/95 (37.9)	
Municipality of residence		
Puerto Berrio	82/222 (36.9)	0.437
Cimitarra	8/16 (50.0)	
Maceo	8/14 (57.1)	
Puerto Nare	4/7 (57.1)	
Others	5/12 (41.7)	
Place of enrollment		
Consultation	11/21 (52.4)	0.29
Emergency	68/186 (36.6)	
Hospitalization	28/64 (43.8)	
Place of residence		
Rural	56/148 (37.8)	0.543
Urban	51/123 (41.5)	
Workplace		
No work	8/15 (53.3)	0.415
Rural	44/121 (36.4)	
Urban	55/135 (40.7)	
Type of health insurance		
Contributory	24/95 (25.3)	0.002
Subsidized	62/128 (48.4)	
Special or exception	21/48 (43.8)	
Occupation		
Veterinarian/farmer	11/22 (50.0)	0.082
Agriculturist	4/9 (44.4)	
Health personnel	0/6 (.0)	
Military or police	20/41 (48.8)	
Other	72/193 (37.3)	
Cats in residence and at work		
Yes	65/156 (41.7)	0.392
No	42/115 (36.5)	
Dogs in residence and at work		
Yes	67/162 (41.4)	0.442
No	40/109 (36.7)	
Cows in residence and at work		
Yes	28/64 (43.8)	0.400
No	78/206 (37.9)	
Pigs in residence and at work		
Yes	17/35 (48.6)	0.239
No	90/236 (38.1)	
Goats in residence and at work		
Yes	3/9 (33.3)	1.000
No	104/262 (39.7)	
Sheep in residence and at work		
Yes	2/8 (25.0)	0.487
No	104/262 (39.7)	
Chickens in residence and at work		
Yes	38/86 (44.2)	0.280
No	69/185 (37.3)	
Horses in residence and at work		
Yes	26/65 (40.0)	0.922
No	81/206 (39.3)	
Do pets sleep indoors?		
Yes	49/111 (44.1)	0.191
No	58/160 (36.3)	
Has any animal had an abortion at the residence?		
Yes	7/19 (36.8)	0.807
No	100/252 (39.7)	
Has any animal had an abortion at work?		
Yes	13/21 (61.9)	0.029
No	94/250 (37.6)	
Exposed to animal births in the last six months		
Yes	18/43 (41.9)	0.728
No	89/228 (39.0)	
Consume boiled or potable water		
Yes	47/128 (36.7)	0.353
No	60/142 (42.3)	
Hand washing before eating or preparing food		
Yes	92/219 (42.0)	0.081
No	15/52 (28.8)	
Raw milk consumption		
Yes	17/42 (40.5)	0.886
No	90/229 (39.3)	
Raw meat consumption or three quarters		
Yes	16/40 (40.0)	0.942
No	91/231 (39.4)	
Preparation of products derived from raw milk		
Yes	21/60 (35.0)	0.421
No	86/211 (40.8)	
Consumption of raw milk derivatives		
Yes	98/249 (39.4)	0.887
No	9/22 (40.9)	
Has a history of visiting farms with animals in the last two months?		
Yes	42/118 (35.6)	0.250
No	65/153 (42.5)	
Another family member is presenting AFI		
Yes	28/83 (33.7)	0.198
No	79/188 (42.0)	
Has a tick-bitten history of ever?		
Yes	94/238 (39.5)	0.991
No	13/33 (39.4)	
Ticks or immature forms of ticks in residence or at work		
Yes	94/238 (39.5)	0.991
No	13/33 (39.4)	
Blood transfusion		
Yes	6/15 (40.0)	0.952
No	100/255 (39.2)	
Travel in the last six months		
Yes	54/142 (38.0)	0.607
No	53/129 (41.1)	
Direct contact with animals at work		
Yes	26/59 (44.1)	0.415
No	81/212 (38.2)	

^1^ The real-time PCR results performed to detect *C. burnetii,* plus the serologic detection of phase I and II IgG antibodies defined as the outcome variable.

**Table 2:** Clinical characteristics of the patients according to the results of tests for the detection of *Coxiella burnetii*.

Clinical Features	Results of Tests for the Detection of *C. burnetii*				
	Negative N = 164	PCR+ Serology+ N = 18	PCR+ Serology− N = 36	PCR- Serology+ N = 53	*p*-Value
	n %	n %	n %	n %	
Shaking chills	155 (94.5)	16 (88.9)	33 (91.7)	47 (88.7)	0.555
Profuse sweating	110 (67.1)	11 (61.1)	22 (62.9)	27 (50.9)	0.212
Sickness	116 (70.7)	10 (55.6)	25 (69.4)	37 (71.2)	0.607
Fatigue	148 (90.2)	17 (94.4)	31 (86.1)	44 (83.0)	0.325
Anorexia	142 (86.6)	16 (88.9)	32 (88.9)	41 (77.4)	0.331
Myalgia	140 (85.4)	16 (88.9)	30 (83.3)	45 (84.9)	0.960
Arthralgia	128 (78.0)	14 (77.8)	27 (75.0)	35 (66.0)	0.366
Headache	149 (90.9)	18 (100.0)	33 (91.7)	46 (86.8)	0.271
Diarrhea	55 (33.5)	4 (22.2)	12 (33.3)	23 (43.4)	0.371
Nauseous	75 (45.7)	12 (66.7)	19 (52.8)	23 (43.4)	0.306
Conjunctivitis	98 (59.8)	12 (66.7)	19 (52.8)	27 (50.9)	0.528
Maculopapular rash	43 (26.4)	3 (16.7)	14 (38.9)	15 (28.8)	0.321
Lymphadenopathy: cervical	10 (6.1)	0 (.0)	0 (.0)	1 (1.9)	0.105
Lymphadenopathy: axillary	5 (3.0)	1 (5.6)	2 (5.6)	4 (7.5)	0.685
Lymphadenopathy: inguinal	10 (6.1)	0 (.0)	3 (8.3)	5 (9.4)	0.428
Lymphadenopathy: epitrochlear	2 (1.2)	0 (.0)	0 (.0)	0 (.0)	0.762
Facial paralysis	5 (3.0)	0 (.0)	0 (.0)	0 (.0)	0.200
Cough	57 (34.8)	2 (11.1)	11 (30.6)	15 (28.3)	0.208
Expectoration	15 (9.1)	2 (11.1)	4 (11.1)	6 (11.3)	0.959
Dyspnea	26 (15.9)	4 (22.2)	7 (19.4)	7 (13.2)	0.772
Nasal congestion	15 (9.1)	5 (27.8)	5 (13.9)	6 (11.3)	0.252
Rhinorrhea	13 (7.9)	3 (16.7)	2 (5.6)	4 (7.5)	0.677
**Physical exam**					
Choluria	63 (38.4)	4 (22.2)	13 (36.1)	15 (28.3)	0.363
Jaundice	7 (4.3)	2 (11.1)	2 (5.6)	3 (5.7)	0.784
Right-upper-quadrant pain	60 (36.6)	8 (44.4)	13 (36.1)	9 (17.0)	0.040
Pruritus	22 (13.4)	2 (11.1)	8 (22.2)	2 (3.8)	0.065
Retro eye pain	41 (25.2)	9 (50.0)	9 (25.0)	13 (24.5)	0.146
Hepatomegaly	2 (1.2)	0 (.0)	0 (.0)	1 (1.9)	1.000
Splenomegaly	2 (1.2)	0 (.0)	0 (.0)	2 (3.8)	0.508
Exanthema	28 (17.1)	2 (11.1)	11 (30.6)	10 (18.9)	0.233
Petechiae	12 (7.3)	2 (11.1)	3 (8.6)	4 (7.5)	0.979
Ecchymosis	3 (1.8)	0 (.0)	0 (.0)	1 (1.9)	0.884
Bleeding	7 (4.3)	0 (.0)	3 (8.3)	0 (.0)	0.073
Conjunctivitis	51 (31.1)	9 (50.0)	10 (27.8)	14 (26.4)	0.292
**Clinical examination**					
Jaundice	3 (1.8)	0 (.0)	0 (.0)	3 (5.7)	0.230
Choluria	29 (17.7)	0 (.0)	5 (13.9)	7 (13.2)	0.237
Right-upper-quadrant pain	52 (31.7)	4 (22.2)	11 (30.6)	8 (15.1)	0.115
Altered consciousness	1 (.6)	0 (.0)	0 (.0)	2 (3.8)	0.201

**Table 3:** Morphological and molecular identifications of ticks, frequency of infestation, and natural infection by molecular detection of ticks.

Site of Sampling	Sampled Animals ^1^/Sampling of Immature Stages ^2^	Immature Stages Obtained ^3^ N = 95 n (%)	Sampled Animals N = 138 n/N (%)	Infested Animals N = 16 n (%)		Tick Identification ^4^		Detected Microorganisms		
						*R. sanguineus* s.l. N = 173 n (%)	*R. microplus* N = 18 n (%)	*Ehrlichia* n (%)	*Anaplasma* n (%)	*Coxiella* n (%)
**10015**	CFFW/Not	0 (0.0)	47 (34.1)	Canines	2 (12.5)	23 (13.3)	0 (0.0)	9 (39.1)	0 (0.0)	0 (0.0)
**10027**	CFFW/Not	0 (0.0)	5 (3.6)	Canines	2 (12.5)	41 (23.7)	0 (0.0)	1 (2.4)	0 (0.0)	5 (12.2)
**10027 ^5^**	C/Yes	0 (0.0)	2 (1.4)	Canines	1 (6.3)	1 (0.6)	0 (0.0)	0 (0.0)	0 (0.0)	0 (0.0)
**10020**	CF/Not	0 (0.0)	8 (5.8)	Canines	3 (18.6)	52 (30.1)	0 (0.0)	15 (28.9)	1 (1.9)	9 (17.3)
**10001**	CBEFFW/Yes	95 (100)	10 (7.2)	Canines	1 (6.3)	26 (15.0)	0 (0.0)	4 (15.4)	1 (3.8)	3 (11.5)
**10083**	C/Not	0 (0.0)	2 (1.4)	Canines	1 (6.3)	4 (2.3)	0 (0.0)	0 (0.0)	0 (0.0)	0 (0.0)
**10198**	CEFFW/Not	0 (0.0)	10 (7.2)	Canines	2 (12.5)	8 (4.6)	0 (0.0)	0 (0.0)	0 (0.0)	2 (25.0)
**10227**	CBFW/Yes	0 (0.0)	18 (13)	Canines	2 (12.5)	18 (10.4)	0 (0.0)	0 (0.0)	0 (0.0)	1 (5.6)
		0 (0.0)		Bovines	2 (12.5)	0 (0.0)	18 (100)	7 (38.9)	6 (33.3)	0 (0.0)
**10094**	/Not	0 (0.0)	0 (0.0)	0 (0.0)		0 (0.0)	0 (0.0)	0 (0.0)	0 (0.0)	0 (0.0)
**10082**	C/Not	0 (0.0)	2 (1.4)	0 (0.0)		0 (0.0)	0 (0.0)	0 (0.0)	0 (0.0)	0 (0.0)
**10131**	C/Not	0 (0.0)	1 (0.8)	0 (0.0)		0 (0.0)	0 (0.0)	0 (0.0)	0 (0.0)	0 (0.0)
**10163**	/Not	0 (0.0)	0 (0.0)	0 (0.0)		0 (0.0)	0 (0.0)	0 (0.0)	0 (0.0)	0 (0.0)
**10205**	FW/Not	0 (0.0)	16 (11.6)	0 (0.0)		0 (0.0)	0 (0.0)	0 (0.0)	0 (0.0)	0 (0.0)
**10242**	CF/Not	0 (0.0)	3 (2.2)	0 (0.0)		0 (0.0)	0 (0.0)	0 (0.0)	0 (0.0)	0 (0.0)
**10243**	CFW/Not	0 (0.0)	12 (8.7)	0 (0.0)		0 (0.0)	0 (0.0)	0 (0.0)	0 (0.0)	0 (0.0)
**10263**	CF/Not	0 (0.0)	2 (1.4)	0 (0.0)		0 (0.0)	0 (0.0)	0 (0.0)	0 (0.0)	0 (0.0)

^1^ Sampled animals: C: canines, B: bovines, E: equines, F: felines, FW: fowls. ^2^ Sampling at immature stages using the dragging technique. ^3^ Only larvae were obtained using the dragging technique. ^4^ Morphological identifications verified with the COI molecular marker. ^5^ Workplace.

**Table 4:** A multivariate model of variables associated with *C. burnetii*.

Features	cPR ^1^ (95% CI)	*p*-value	aPR ^2^ (95% CI)	*p*-value
Demographic				
Social security				
Contributory	Reference	-	Reference	-
Subsidized	1.91 (1.57–2.32)	0.000	2.08 (1.67–2.59)	0.000
Special or exception	1.73 (1.39–2.14)	0.000	2.06 (1.61–2.63)	0.000
Epidemiological				
Abortions at work	1.64 (1.35–1.99)	0.000	1.81 (1.44–2.28)	0.000
Pets sleep in the house	1.21 (1.02–1.44)	0.022	1.45 (1.24–1.71)	0.000
Symptoms in family members	0.80 (0.70–0.92)	0.022	0.78 (0.65–0.94)	0.010
Clinical				
Sweating	0.76 (0.72–0.80)	0.000	0.79 (0.69–0.91)	0.001
Chill	0.69 (0.51–0.94)	0.018	0.75 (0.63–0.89)	0.001
Congestion	1.36 (0.96–1.91)	0.078	1.66 (1.24–2.23)	0.001
Arthralgia	0.80 (0.67–0.95)	0.013	0.75 (0.63–0.91)	0.003
Jaundice	1.28 (0.99–1.66)	0.058	1.58 (1.02–2.44)	0.037
Petechiae	1.10 (0.78–1.53)	0.575	1.32 (0.98–1.77)	0.061
Choluria (physical exam)	0.70 (0.57–0.86)	0.001	0.70 (0.29–0.38)	0.002

^1^ Crude prevalence ratio; ^2^ adjusted prevalence ratio.

## Data Availability

The datasets generated for this study are available upon request from the corresponding author. *C. burnetii* sequences MN540438, MN540439, MN540440, MN540441, OM474972, and OM474973; CLB from *Rhipicephalus sanguineus* sequences OM475004, OM475005, OM475006, OM475007, OM475008, OM475009, OM475010, OM475011, and OM475012; *Anaplasma platys* sequences OM475363 and OM475364; *Anaplasma marginale* sequence OM475362; and uncultured *Ehrlichia* sp. sequence OM475361.

## Supplementary Materials

The following supporting information can be downloaded at: https://www.mdpi.com/article/10.3390/pathogens11101090/s1, Table S1: Demographic and epidemiological characteristics according to the serological results of the bacteria studied; Table S2: Selected serology kits for the detection of antibody anti- IgG and primers for the molecular detection of microorganisms of the genera *Rickettsia, Anaplasma, Borrelia, Ehrlichia, Coxiella*, and *Francisella*.
