# Supplementary material for: Tick-Borne-Agents Detection in Patients with Acute Febrile Syndrome and Ticks from Magdalena Medio, Colombia
Source: Pathogens. 2022 Sep 24;11(10):1090. doi: 10.3390/pathogens11101090 (PMC9611641; doi:10.3390/pathogens11101090)
Supplement: Supplementary file 1 [file pathogens-11-01090-s001.zip › pathogens-1887166-supplementary.pdf]

Supplementary Table S1. Demographic and epidemiological characteristics according to the serological results of the bacteria studied.

Demographic/epidemiological characteristics	*Francisella* n/N (%)	*Anaplasma* n/N (%)	*Ehrlichia* n/N (%)	*R. rickettsii* n/N (%)	*R. typhi* n/N (%)	*Borrelia* n/N (%)
**Sex**						
**Male**	73/176 (41.5)	24/176 (13.6)	47/176 (26.7)	61/176 (34.7)	27/176 (15.3)	15/176 (8.5)
**Female**	34/95 (35.8)	15/95 (15.8)	26/95 (27.4)	24/95 (25.3)	15/95 (15.8)	3/95 (3.2)
**Municipality of residence**						
**Puerto Berrio**	88/222 (39.6)[a]	27/222 (12.2)[a]	56/222 (25.2)	70/222 (31.5)	32/222 (14.4)	13/222 (5.9)
**Cimitarra**	9/16 (56.3)	6/16 (37.5)	8/16 (50.0)	2/16 (12.5)	3/16 (18.8)	1/16 (6.3)
**Maceo**	4/14 (28.6)	1/14 (7.1)	4/14 (28.6)	5/14 (35.7)	3/14 (21.4)	0/14 (.0)
**Puerto Nare**	3/7 (42.9)	3/7 (42.9)	3/7 (42.9)	4/7 (57.1)	1/7 (14.3)	2/7 (28.6)
**Others**	3/12 (25.0)	2/12 (16.7)	2/12 (16.7)	4/12 (33.3)	3/12 (25.0)	2/12 (16.7)
**Place of enrollment**						
**Consultation**	3/21 (14.3)	3/21 (14.3)	6/21 (28.6)	5/21 (23.8)	4/21 (19.0)	1/21 (4.8)
**Emergency**	84/186 (45.2)	25/186 (13.4)	46/186 (24.7)	62/186 (33.3)	29/186 (15.6)	11/186 (5.9)
**Hospitalization**	20/64 (31.3)	11/64 (17.2)	21/64 (32.8)	18/64 (28.1)	9/64 (14.1)	6/64 (9.4)
**Place of residence**						
**Rural**	59/148 (39.9)	19/148 (12.8)	41/148 (27.7)	40/148 (27.0)	21/148 (14.2)	7/148 (4.7)
**Urban**	48/123 (39.0)	20/123 (16.3)	32/123 (26.0)	45/123 (36.6)	21/123 (17.1)	11/123 (8.9)
**Workplace**						
**No work**	4/15 (26.7)	2/15 (13.3)	7/15 (46.7)	5/15 (33.3)	1/15 (6.7)	0/15 (.0)
**Rural**	49/121 (40.5)	15/121 (12.4)	32/121 (26.4)	31/121 (25.6)	18/121 (14.9)	5/121 (4.1)
**Urban**	54/135 (40.0)	22/135 (16.3)	34/135 (25.2)	49/135 (36.3)	23/135 (17.0)	13/135 (9.6)
**Type of social security**						
**Contributory**	44/95 (46.3)	11/95 (11.6)	26/95 (27.4)	30/95 (31.6)	10/95 (10.5)	6/95 (6.3)
**Subsidized**	46/128 (35.9)	21/112 (18.8)	38/128 (29.7)	40/128 (31.3)	25/128 (19.5)	10/128 (7.8)
**Special or exception**	17/48 (35.4)	5/48 (10.4)	9/48 (18.8)	15/48 (31.3)	7/48 (14.6)	2/48 (4.2)

**Ethnicity**						
**Afrocolombian**	0/1 (.0)	0/1 (.0)	0/1 (.0)	0/1 (.0)	0/1 (.0)	0/1 (.0)
**ROM (gypsy)**	2/5 (40.0)	1/5 (20.0)	1/5 (20.0)	2/5 (40.0)	1/5 (20.0)	2/5 (40.0)
**Other**	105/265 (39.6)	38/265 (14.3)	72/265 (27.2)	83/265 (31.3)	41/265 (15.5)	16/265 (6.0)
**Occupation**						
**Veterinarian/Farmer**	11/22 (50.0)	4/22 (18.2)	8/22 (36.4)[a]	11/22 (50.0)	2/22 (9.1)	2/22 (9.1)
**Zootechnician**	0/0 (.0)	0/0 (.0)	0/0 (.0)	0/0 (.0)	0/0 (.0)	0/0 (.0)
**Agriculturist**	2/9 (22.2)	3/9 (33.3)	6/9 (66.7)	3/9 (33.3)	4/9 (44.4)	1/9 (11.1)
**Health personnel**	4/6 (66.7)	0/6 (.0)	1/6 (16.7)	0/6 (.0)	0/6 (.0)	0/6 (.0)
**Military or Police**	13/41 (31.7)	3/41 (7.3)	7/41 (17.1)	12/41 (29.3)	7/41 (17.1)	3/41 (7.3)
**Other**	77/193 (39.9)	29/193 (15.0)	51/193 (26.4)	59/193 (30.6)	29/193 (15.0)	12/193 (6.2)
**Cats in residence and work**						
**Yes**	62/156 (39.7)	21/156 (13.5)	50/156 (32.1)[a]	56/156 (35.9)	26/156 (16.7)	10/156 (6.4)
**No**	45/115 (39.1)	18/115 (15.7)	23/115 (20.0)	29/115 (25.2)	16/115 (13.9)	8/115 (7.0)
**Dogs in residence and work**						
**Yes**	65/162 (40.1)	22/162 (13.6)	46/162 (28.4)	55/162 (34.0)	25/162 (15.4)	10/162 (6.2)
**No**	42/109 (38.5)	17/109 (15.6)	27/109 (24.8)	30/109 (27.5)	17/109 (15.6)	8/109 (7.3)
**Cows in residence and work**						
**Yes**	27/64 (42.2)	8/64 (12.5)	21/64 (32.8)	23/64 (35.9)	7/64 (10.9)	8/64 (12.5)[a]
**No**	80/206 (38.8)	31/206 (15.0)	52/206 (25.2)	61/206 (29.6)	34/206 (16.5)	10/206 (4.9)
**Pigs in residence and work**						
**Yes**	15/35 (42.9)	7/35 (20.0)	13/35 (37.1)	12/35 (34.3)	6/35 (17.1)	2/35 (5.7)
**No**	92/236 (39.0)	32/236 (13.6)	60/236 (25.4)	73/236 (30.9)	36/236 (15.3)	16/236 (6.8)
**Goats in residence and work**						
**Yes**	6/9 (66.7)	1/9 (11.1)	5/9 (55.6)	4/9 (44.4)	1/9 (11.1)	0/9 (.0)
**No**	101/262 (38.5)	38/262 (14.5)	68/262 (26.0)	81/262 (30.9)	41/262 (15.6)	18/262 (6.9)
**Sheep in residence and work**						
**Yes**	5/8 (62.5)	2/8 (25.0)	5/8 (62.5)[a]	3/8 (37.5)	1/8 (12.5)	0/8 (.0)
**No**	101/262 (38.5)	36/262 (13.7)	67/262 (25.6)	82/262 (31.3)	41/262 (15.6)	18/262 (6.9)

**Hens in residence and work**						
**Yes**	34/86 (39.5)	17/86 (19.8)	24/86 (27.9)	34/86 (39.5)[a]	10/86 (11.6)	9/86 (10.5)
**No**	73/185 (39.5)	22/185 (11.9)	49/185 (26.5)	51/185 (27.6)	32/185 (17.3)	9/185 (4.9)
**Horses in residence and work**						
**Yes**	30/65 (46.2)	9/65 (13.8)	21/65 (32.3)	24/65 (36.9)	9/65 (13.8)	8/65 (12.3)[a]
**No**	77/206 (37.4)	30/206 (14.6)	52/206 (25.2)	61/206 (29.6)	33/206 (16.0)	10/206 (4.9)
**Do pets sleep indoors?**						
**Yes**	49/111 (44.1)	13/111 (11.7)	29/111 (26.1)	38/111 (34.2)	19/111 (17.1)	4/111 (3.6)
**No**	58/160 (36.3)	26/160 (16.3)	44/160 (27.5)	47/160 (29.4)	23/160 (14.4)	14/160 (8.8)
**Do any animal abortions at the residence?**						
**Yes**	8/19 (42.1)[a]	1/19 (5.3)	3/19 (15.8)	7/19 (36.8)	2/19 (10.5)	1/19 (5.3)
**No**	99/252 (39.4)	38/252 (15.1)	70/252 (27.8)	78/252 (31.0)	40/252 (15.9)	17/252 (6.7)
**Do any animal abortions at work?**						
**Yes**	14/21 (66.7)	3/21 (14.3)	8/21 (38.1)	9/21 (42.9)	2/21 (9.5)	3/21 (14.3)
**No**	93/250 (37.2)	36/250 (14.4)	65/250 (26.0)	76/250 (30.4)	40/250 (16.0)	15/250 (6.0)
**Exposed to abortions in the last six months**						
**Yes**	19/43 (44.2)	7/43 (16.3)	13/43 (30.2)	17/43 (39.5)	7/43 (16.3)	4/43 (9.3)
**No**	88/228 (38.6)	32/228 (14.0)	60/228 (26.3)	68/228 (29.8)	35/228 (15.4)	14/228 (6.1)
**Consume boiled or potable water**						
**Yes**	51/128 (39.8)	28/128 (21.9)[a]	39/128 (30.5)	38/128 (29.7)	17/128 (13.3)	7/128 (5.5)
**No**	55/142 (38.7)	11/142 (7.7)	34/142 (23.9)	47/142 (33.1)	25/142 (17.6)	11/142 (7.7)
**Hand washing before eating or preparing food**						
**Yes**	90/219 (41.1)	37/219 (16.9)[a]	64/219 (29.2)	67/219 (30.6)	34/219 (15.5)	15/219 (6.8)
**No**	17/52 (32.7)	2/52 (3.8)	9/52 (17.3)	18/52 (34.6)	8/52 (15.4)	3/52 (5.8)
**Raw milk consumption**						
**Yes**	13/42 (31.0)	3/42 (7.1)	9/42 (21.4)	18/42 (42.9)	6/42 (14.3)	4/42 (9.5)
**No**	94/229 (41.0)	36/229 (15.7)	64/229 (27.9)	67/229 (29.3)	36/229 (15.7)	14/229 (6.1)
**Raw meat consumption or three quarters**						

**Yes**	20/40 (50.0)	4/40 (10.0)	14/40 (35.0)	10/40 (25.0)	6/40 (15.0)	0/40 (.0)
**No**	87/231 (37.7)	35/231 (15.2)	59/231 (25.5)	75/231 (32.5)	36/231 (15.6)	18/231 (7.8)
**Preparation of products derived from raw milk**						
**Yes**	22/60 (36.7)	10/60 (16.7)	17/60 (28.3)	18/60 (30.0)	7/60 (11.7)	5/60 (8.3)
**No**	85/211 (40.3)	29/211 (13.7)	56/211 (26.5)	67/211 (31.8)	35/211 (16.6)	13/211 (6.2)
**Consumption of raw milk derivatives**						
**Yes**	99/249 (39.8)	35/249 (14.1)	65/249 (26.1)	78/249 (31.3)	38/249 (15.3)	16/249 (6.4)
**No**	8/22 (36.4)	4/22 (18.2)	8/22 (36.4)	7/22 (31.8)	4/22 (18.2)	2/22 (9.1)
**Rodents at the residence**						
**Yes**	73/179 (40.8)	22/179 (12.3)	41/179 (22.9)[a]	57/179 (31.8)	28/179 (15.6)	12/179 (6.7)
**No**	34/92 (37.0)	17/92 (18.5)	32/92 (34.8)	28/92 (30.4)	14/92 (15.2)	6/92 (6.5)
**Cockroaches at the residence**						
**Yes**	88/220 (40.0)	31/220 (14.1)	57/220 (25.9)	69/220 (31.4)	33/220 (15.0)	13/220 (5.9)
**No**	19/51 (37.3)	8/51 (15.7)	16/51 (31.4)	16/51 (31.4)	9/51 (17.6)	5/51 (9.8)
**Flies at the residence**						
**Yes**	86/223 (38.6)	33/223 (14.8)	59/223 (26.5)	72/223 (32.3)	36/223 (16.1)	15/223 (6.7)
**No**	21/48 (43.8)	6/48 (12.5)	14/48 (29.2)	13/48 (27.1)	6/48 (12.5)	3/48 (6.3)
**Fleas at the residence**						
**Yes**	25/70 (35.7)	5/70 (7.1)[a]	16/70 (22.9)	21/70 (30.0)	6/70 (8.6)	6/70 (8.6)
**No**	82/201 (41.0)	34/201 (16.9)	57/201 (28.4)	64/201 (31.8)	36/201 (17.9)	12/201 (6.0)
**Mites at the residence**						
**Yes**	23/54 (42.6)	7/54 (13.0)	12/54 (22.2)	18/54 (33.3)	12/54 (22.2)	3/54 (5.6)
**No**	84/217 (38.7)	32/217 (14.7)	61/217 (28.1)	67/217 (30.9)	30/217 (13.8)	15/217 (6.9)
**Has a history of visiting farms with animals in the last two months?**						
**Yes**	45/118 (38.1)	16/118 (13.6)	37/118 (31.4)	39/118 (33.1)	15/118 (12.7)	8/118 (6.8)
**No**	62/153 (40.5)	23/153 (15.0)	36/153 (23.5)	46/153 (30.1)	27/153 (17.6)	10/153 (6.5)
**Other families member is presenting AFI?**						
**Yes**	39/83 (47.0)	10/83 (12.0)	21/83 (25.3)	22/83 (26.5)	15/83 (18.1)	7/83 (8.4)

**No**	68/188 (36.2)	29/188 (15.4)	52/188 (27.7)	63/188 (33.5)	27/188 (14.4)	11/188 (5.9)
**Mosquito bites in the past month**						
**Yes**	100/253 (39.5)	37/253 (14.6)	66/253 (26.1)	83/253 (32.8)	41/253 (16.2)	18/253 (7.1)
**No**	7/18 (38.9)	2/18 (11.1)	7/18 (38.9)	2/18 (11.1)	1/18 (5.6)	0/18 (.0)
**Has a tick bitten a history of ever?**						
**Yes**	92/238 (38.7)	33/238 (13.9)	69/238 (29.0)[a]	74/238 (31.1)	37/238 (15.5)	18/238 (7.6)
**No**	15/33 (45.5)	6/33 (18.2)	4/33 (12.1)	11/33 (33.3)	5/33 (15.2)	0/33 (.0)
**Ticks or immature forms of ticks in residence or work**						
**Yes**	92/238 (38.7)	33/238 (13.9)	69/238 (29.0)[a]	74/238 (31.1)	37/238 (15.5)	18/238 (7.6)
**No**	15/33 (45.5)	6/33 (18.2)	4/33 (12.1)	11/33 (33.3)	5/33 (15.2)	0/33 (.0)
**Blood transfusion?**						
**Yes**	6/15 (40.0)	4/15 (26.7)	3/15 (20.0)	6/15 (40.0)	4/15 (26.7)	3/15 (20.0)
**No**	101/255 (39.6)	35/255 (13.7)	70/255 (27.5)	78/255 (30.6)	38/255 (14.9)	15/255 (5.9)
**Travel in the last six months**						
**Yes**	54/142 (38.0)	21/142 (14.8)	44/142 (31.0)	41/142 (28.9)	18/142 (12.7)	10/142 (7.0)
**No**	53/129 (41.1)	18/129 (14.0)	29/129 (22.5)	44/129 (34.1)	24/129 (18.6)	8/129 (6.2)
**Direct contact with animals at work**						
**Yes**	29/59 (49.2)	9/59 (15.3)	22/59 (37.3)[a]	21/59 (35.6)	7/59 (11.9)	6/59 (10.2)
**No**	78/212 (36.8)	30/212 (14.2)	51/212 (24.1)	64/212 (30.2)	35/212 (16.5)	12/212 (5.7)

[a] *p*-value≤0.05

Supplementary Table S2. Selected serology kits for the detection of antibody anti-IgG and primers for the molecular detection of microorganisms of the genera *Rickettsia, Anaplasma, Borrelia, Ehrlichia, Coxiella,* and *Francisella*

Microorganism	Serology		Molecular detection			
	Kit	Brand	Primers	Target gene	Type of PCR	Ref
Anaplasma	*Anaplasma/Ehrlichia* IgG MIF Kit	FULLER LABORATORIES	EC-SYBRF<br>EC-SYBRR	16S rRNA	Real time PCR with EvaGreen	[48]
Ehrlichia	*Anaplasma/Ehrlichia* IgG MIF Kit	FULLER LABORATORIES	DSB-330<br>DSB-728	Disulfide bond formation protein	Conventional PCR	[49]
Rickettsia	RICKETTSIA IFA IGG[a]	DIASORIN	CS-F<br>CS-R<br>CS-P	Citrate synthase (gltA)	Real time PCR with probe	[50]
Coxiella	Q FEVER IFA IGG[a]	DIASORIN	IS1111-f<br>IS1111-r<br>IS1111-p	Insertion sequence	Real time PCR with probe	[51]
			Cox16SF1<br>Cox16SR2	16S rRNA	Conventional nested PCR	[68]
			Cox16SF1<br>Cox16SR1			
Borrelia	BORRELIA BURGDORFERI IGG (LYME) RECOMBINANT ANTIGENS + LIQUOR	NOVATEC	Bor16S-f<br>Bor16S-r	16S rRNA	Conventional PCR	[52]
Francisella	*Francisella tularensis* IgG MIF Kit	FULLER LABORATORIES	Fra16S-f<br>Fra16S-r	16S rRNA	Conventional PCR	[53]

[a] These kits are FDA approved for diagnostics.

References

[48] G. Seong, Y.-J. Han, J.-B. Chae, J.-S. Chae, D.-H. Yu, Y.-S. Lee, J. Park, B.-K. Park, J.-G. Yoo, K.-S. Choi, Detection of Anaplasma sp. in Korean Native Goats (Capra aegagrus hircus) on Jeju Island, Korea, Korean J Parasitol. 53 (2015) 765–769. https://doi.org/10.3347/kjp.2015.53.6.765.

[49] C.K. Doyle, M.B. Labruna, E.B. Breitschwerdt, Y.-W. Tang, R.E. Corstvet, B.C. Hegarty, K.C. Bloch, P. Li, D.H. Walker, J.W. McBride, Detection of Medically Important Ehrlichia by Quantitative Multicolor TaqMan Real-Time Polymerase Chain Reaction of the dsb Gene, J Mol Diagn. 7 (2005) 504–510.

[50] G. Madeddu, F. Mancini, A. Caddeo, A. Ciervo, S. Babudieri, I. Maida, M.L. Fiori, G. Rezza, M.S. Mura, Rickettsia monacensis as Cause of Mediterranean Spotted Fever–like Illness, Italy, Emerg Infect Dis. 18 (2012) 702–704. https://doi.org/10.3201/eid1804.111583.

[51] G.B. Howe, B.M. Loveless, D. Norwood, P. Craw, D. Waag, M. England, J.R. Lowe, B.C. Courtney, M.L. Pitt, D.A. Kulesh, Real-time PCR for the early detection and quantification of Coxiella burnetii as an alternative to the murine bioassay, Mol. Cell. Probes. 23 (2009) 127–131. https://doi.org/10.1016/j.mcp.2009.01.004.

[52] S.H. Lee, V.S. Vigliotti, J.S. Vigliotti, W. Jones, S. Pappu, Increased sensitivity and specificity of Borrelia burgdorferi 16S ribosomal DNA detection, Am. J. Clin. Pathol. 133 (2010) 569–576. https://doi.org/10.1309/AJCPI72YAXRHYHEE.

[53] S.J. Dergousoff, N.B. Chilton, Association of Different Genetic Types of Francisella-Like Organisms with the Rocky Mountain Wood Tick (Dermacentor andersoni) and the American Dog Tick (Dermacentor variabilis) in Localities Near Their Northern Distributional Limits, Appl Environ Microbiol. 78 (2012) 965–971. https://doi.org/10.1128/AEM.05762-11.

[68] O. Duron, E. Jourdain, K.D. McCoy, Diversity and global distribution of the Coxiella intracellular bacterium in seabird ticks, Ticks Tick Borne Dis. 5 (2014) 557–563. https://doi.org/10.1016/j.ttbdis.2014.04.003.
